# Topical application of cervix with hyaluronan improves fertility in goats inseminated with frozen-thawed semen

**DOI:** 10.5713/ajas.20.0284

**Published:** 2020-08-24

**Authors:** Sukanya Leethongdee, Anone Thuangsanthia, Muhammad Khalid

**Affiliations:** 1 The Reproduction in Domestic Animal Research Unit, Faculty of Veterinary Science, Mahasarakham University, Mahasarakham 44000, Thailand; 2 Embryo Transfer Technology Development and Reproductive Cell Research Center, Department of Livestock Development, Nakorn Ratchasima 30130, Thailand; 3 Department of Pathobiology and Population Sciences, The Royal Veterinary College, University of London, Hertfordshire AL9 7TA, United Kingdom

**Keywords:** Goat Cervix, Hyaluronan, Cervical Insemination, Frozen-thawed Semen

## Abstract

**Objective:**

Artificial insemination plays an important role in genetic improvement in the goat farming system. The aim of this study was to investigate the effect of cervical application of hyaluronan (HA) on the fertility in goats after cervical artificial insemination using frozen-thawed (F-T) semen.

**Methods:**

After oestrous synchronisation with progesterone sponges and pregnant mare serum gonadotropin injection, both nulli- and multi-parous goats, were randomly allocated to 2 groups, and were inseminated with 0.25 mL of F-T semen (150×10^6^ spermatozoa) twice at 52 h and 56 h after sponge removal. Prior to the insemination, goats in Group 1 only were given topical cervical HA application at 48 h after sponge removal. Site of insemination was recorded as os-cervix or intra-cervix or intra-uterus. Pregnancy was tested ultrasonographically 42 days after insemination. The data on pregnancy rates and percentage of animals according to the site of semen deposition were compared by Chi-square analysis.

**Results:**

The overall pregnancy rate was significantly (p<0.004) higher in goats with prior application to the cervix with HA (63.3%) than without (36.0%). Same pattern was observed in the pregnancy rates of nulli- and multi-parous goats in both the groups. Percentage of nulliparous goats according to the site of insemination in the HA group did not differ between first and the second insemination. However, in multiparous goats the percentage of animals inseminated intra-cervically was significantly increased (p≤0.05) between the first and the second inseminations.

**Conclusion:**

The results suggest that significantly higher fertility rate in the “HA goats” compared to the “without HA” group was because of deeper insemination facilitated by topical cervical application of HA. The deeper insemination into the cervical canal increase the rate of fertilisation when the cervical artificial insemination is performed.

## INTRODUCTION

Artificial insemination (AI) is practiced to improve the genetics of farm animals including goats [1]. AI is also helpful containing contagious diseases within the herd and reducing the risk of spreading sexually transmitted infections associated with natural mating. Despite many benefits, use of AI is limited in small ruminants. Three types of AI techniques are used in small ruminants: vaginal, cervical and laparoscopic intrauterine insemination. Vaginal insemination involves depositing fresh diluted semen deep in the anterior vagina without any attempt to locate the cervix. It requires large semen doses (400 to 500×10^6^ spermatozoa per insemination) but fertility is quite poor [1–4]. In cervical insemination, semen is deposited at the beginning of the cervix; at the deepest possible intra-cervical site [5,6]. The depth of semen deposition, depends on the morphology of cervical canal and the type of cervical os [7,8] and has an effect on the fertility rates which increase as the depth of insemination into the cervix increases [9–12]. Fertility with cervical insemination using fresh diluted semen is higher than that achieved with vaginal insemination and is commercially acceptable. However, fertility with cervical insemination using frozen-thawed (F-T) semen is quite poor [13–15] and unacceptable at commercial levels.

Both sheep [8] and goat [7] have complex anatomy that limits the passage of an inseminating pipette through the cervix and therefore makes the deeper deposition of semen difficult. This difficulty in cervical passage of the inseminating pipette has been overcome by deposition of the semen directly into the uterus with the help of a laparoscope [16,17]. And higher fertilization rates have been reported in sheep after laparoscopic insemination (92.5%) compared to cervical AI (39.67%) [3,18–20]. Although laparoscopic insemination can provide better fertility with F-T semen, it cannot be effectively used at a mass scale because of its limitations including higher cost and the technical expertise required to perform it. Considering these limitations, it is highly desirable to develop a method for trans-cervical intrauterine AI which could allow the use of F-T semen to be deposited into the uterus via the vagina and cervix. As complex anatomy of the small ruminant such as sheep [8] and goat [7] cervix limits the passage of an inseminating pipette through the cervix, there is a need of cervical relaxation to pass the inseminating pipette and deposit the semen into the uterus. Different substances have been used in the past to relax the cervix including oxytocin [21], estradiol [22], follicle stimulating hormone (FSH), luteinizing hormone (LH), and prostaglandin E2 (PGE2) [23] with variable results [21,22, 24–26].

Hyaluronan is a non-sulphated glycosaminoglycan pres ent in the cervix and its cervical content varies with the stage of the oestrous cycle, being the highest at oestrus [27–29]. Moreover, topical application to cervix with PGE2 increases the cervical content of HA in sheep [30]. These data suggest that HA can play an important role in cervical relaxation. In this respect, topical intra-cervical application of HA 48 h to 50 h after progesterone sponge removal has been reported to increase the cervical penetrability in sheep [26,27]. However, so far, no attempt has been made to inseminate the animals and study their fertility after cervical relaxation with HA. Therefore, the main aim of this study was to investigate the effect of intra-cervical application of HA on the fertility of goats after cervical insemination with F-T semen.

## MATERIALS AND METHODS

### Animal care

The experiment procedure was approved by the Institutional Animal Care and Use Committee at Mahasarakham University Thailand (0008/2552).

### Animal management and oestrous synchronization

The study was conducted at a commercial goat farm during October until April of the following year in Prachuap Khiri Khan Province, Thailand (Latitude: 11° 49′ 15.53″ N, Longitude: 99° 47′ 2.76″ E). The average body weight and body condition score of the goats were 30±2.5 kg and 3.0 to 3.5, respectively. During the experiments, goats were housed indoors on straw bedding, fed with hay *ad libitum* and a commercial concentrate diet having 16% protein, with water always available.

Prior to the experiments all the animals were scanned by ultrasonography (HS-2000 Honda Electronics CO. LTD, Toyohashi, Aichi, Japan) with trans-rectal probe inducer to confirm the healthy reproductive tracts and their non-pregnant status. All the nannies were synchronized to a common day of oestrus using intra-vaginal Progesterone sponges containing 65 mg medroxyprogesterone (Sincro-gest, Laboratorios Ovejero, Leon, Spain) for 12 days. At the time of sponge removal, nannies were injected intramuscularly with 250 IU pregnant mare serum gonadotropin (PMSG; Intervet Thailand Ltd., Bangkok, Thailand). At 24 h and 48 h after the sponge removal, the present of oestrous signs were observed by presenting the Billy goat outside the female goats’s pen, each time for 2 h.

### The experimental design

A total of 110 cross-bred Boer-goats (57 nulliparous and 53 multiparous) were randomly allocated to 2 groups. All the goats were inseminated each time with 0.25 mL F-T semen containing 150×10^6^ spermatozoa. The F-T semen used for all the goats was from the same production batch and the same Billy goat and had a post-thaw progressive motility of 45%. Goats in Group 1 (26 nulliparous and 34 multiparous) and Group 2 (31 nulliparous and 19 multiparous) were inseminated twice at 52 h and 56 h after the sponge removal using cervical AI with or without prior topical application of HA. Topical application of 40mg HA was accomplished at 48h after the sponge removal (group 1 only) as described by Perry et al [26].

### The semen preparation for artificial insemination

A healthy 3 years old Boer Billy goat was used in this study. The fresh semen was collected using the artificial vagina. The collected fresh semen was placed in a 37°C water bath prior to evaluation. Sperm motility, progressive motility and viability were evaluated. The quality of fresh semen was evaluated in which sperm motility, progressive motility and sperm viability were 95%, 80%, and 90%, respectively. After the evaluation, sperm cryopreservation was performed. The seminal plasma was removed by centrifuged at 1,500×g for 10 minutes using Lactate ringer solution with the proportion of semen:Lactatate ringer solution of 1:19. After that the supernatant was discarded leaving the sperm platelet. Then a commercial extender consisting of 34% of One-step (Continental Plastic corp., Delavan, WI, USA), 46% of distill water and 20% of egg yolk was added gently onto the semen platelet at 37°C to make up the semen extender. The concentration of sperm extender was 600×10^6^ spermatozoa/mL. The semen extender was loaded into 0.25 mL straws which made 150×10^6^ spermatozoa per straw. The semen straws were gradually cooled down in 4°C cabinet for 4 hours. After that all semen straws were frozen at 4 cm above the liquid nitrogen level for 13 minutes then were stored in the liquid nitrogen at −196°C. Twenty four hours following the liquid Nitrogen storage, sperm quality was evaluated after thawing. The frozen semen was thawed by placing the frozen semen straw into the 37°C water bath for 30 seconds, then the thawed semen was dropped on 37°C warm glass slide and the sperm motility, progressive motility and viability were evaluated at 400 magnification under phase contrast microscope. In the present study, the F-T semen with sperm motility, progressive motility and sperm viability of 69%, 70%, and 52%, respectively was used for AI.

### The artificial insemination procedure

The rear part of the goat was placed over a rail. The rear end of the goat was firmly and gently restrained by the inseminator’s assistant. The vulva and the surrounding area were cleaned using a disinfectant wipe and let air dried prior to the insertion of the duckbill vaginal speculum which was fitted with a light source to locate the site of the cervical opening. Once the cervical opening was located, cervical artificial insemination (CAI) was performed using the duckbill vaginal speculum lubricated with the non-toxic gel for spermatozoa. The F-T was thawed by placing the straw into the 37°C water bath for 30 seconds after that both ends of the straw were cut letting the F-T semen run into the 1.5 mL tube which placed in the 37°C heated block. The amount of 0.25 mL F-T semen was loaded into the inseminating pipettes which attached with 1 mL syringe. The F-T semen was deposited using an inseminating pipette which was a modified 6F cardiac catheter (diameter = 2.0 mm), 35 cm long, brite tip sheath introducer (Cordis, Johnson and Johnson company, Miami Lakes, FL, USA) attached to a 1 mL tuberculin syringe. The cervical opening was located and then the tip of the inseminating pipette was inserted into the cervical canal with the help of a stylet and was gently pushed through the cervical canal without any force so as to achieve maximum penetrability. Once at place, stylet was removed, and F-T semen was deposited by pushing the plunger of the 1 mL syringe.

The site of semen deposition was recorded for each ani mal. When the F-T semen was deposited at the opening of the cervix the “cervical os” was recorded, or “intra-cervix” was recorded when the inseminating pipette was passed into the cervical canal but no further, then the inseminator deposited the F-T semen in the cervical canal. Lastly the “intra-uterus” was recorded when the inseminating pipette was passed through the cervical canal reaching into the uterine body where the F-T semen was deposited.

### The pregnancy diagnosis

All the goats were scanned to diagnose pregnancy 42 days after the insemination using ultrasonography scanner (HS-2000; Honda Electronics CO., LTD., Japan) with trans-rectal probe inducer (7.5 MHz). The presence of the foetus and foetal membranes ratified the pregnancy.

### Statistical analysis

The pregnancy rates or the site of semen deposition were calculated and compared between the 2 treatment groups using chi-square statistical analysis. The chi-square statistic calculated from the 2×2 crosstab table testing the independence between the two classifications. The statistical package used in this study was SAS University Edition 2018 (SAS Studio Release: 3.8 [Basic Edition] SAS Institute Inc., Cary, NC, USA). The differences between the groups were considered significant at 5% probability (p<0.05).

## RESULTS

### The percentage of pregnancy

The overall pregnancy rate in the goats inseminated with F-T semen was significantly higher (p<0.004) with (63.3%) than without (36.0%) topical application of the cervix with HA (Table 1).

In nulliparous goats, pregnancy rate was significantly higher (p<0.045) with (65.4%) than without (38.7%) topical application of the cervix with HA. The same was true for multiparous goats and the pregnancy rate was significantly higher with (61.8%) than without (31.6%) prior cervical application of HA, respectively (Table 1). There were no differences in the pregnancy rates between the nulliparous and multiparous goats when inseminated with or without topical application of the cervix with HA (Table 1).

### The site of semen deposition

Overall, the topical application of the cervix with HA significantly increased the number/percentage of goats in which semen could be deposited deeper within the reproductive tract. At first insemination (52 h after the sponge removal), regardless of parity, in Group 1, percentage of goats with semen deposited at cervical os, intra-cervix and intra-uterus was 56.7%, 23. 3%, and 20.0%, respectively (Table 2).

At the time of second insemination (56 h after the sponge removal) illustrated that regardless of parity, in group 1 (with cervical topical application of HA) 50% of goats were inseminated at the cervical os, 30% at intra cervix, and 20% were inseminated intra-uterus.

Without topical application of the HA (group 2), at both first and second insemination, semen could be deposited at the cervical os only and none of the goats in this group had semen deposited at intra cervix or intra uterus (Tables 2, 3).

In nulliparous goats, with cervical topical application of HA, both at first and second insemination (52 h and 56 h after sponge removal, respectively), semen was deposited in 69.2% of goats at the os cervix and in 30.77% of goats at the intra-cervix. HA application significantly (p≤0.05) increased the percentage of nulliparous goats in which semen could be deposited intra-cervix compared to those without HA (group 2). However, even with HA application, in none of the nulliparous goats semen could be deposited into the uterus (Tables 2, 3). In multiparous goats, HA application, not only significantly (p≤0.05) increased the percentage of goats that were inseminated intra-cervix but also allowed 35% of animals to be inseminated intra-uterus. In both nulli- and multi-parous goats, at both inseminations, without HA application, semen could only be deposited at the os-cervix and none of the goats were inseminated intra-cervix or intra-uterus (Tables 2, 3).

## DISCUSSION

In trans-cervical intra-uterine artificial insemination (TCAI), an attempt is made to traverse the cervix with the inseminating pipette to deposit semen into the uterus. The deeper insemination of semen inside the uterus helps the spermatozoa to more easily reach the fertilization site (ampulla of the oviduct) which increases the fertilization rate [18]. TCAI is commonly practiced, in species like cattle and buffaloes, without any problem as the inseminating pipette passes easily through the cervical canal at the time of oestrus. However, small ruminants such as sheep [8] and goats [7] have unusual cervical anatomy, and the presence of cervical folds in these species makes it difficult to pass the inseminating pipette into the uterus via the cervical canal [23]. It is for these reasons that in sheep and goats, semen is mostly deposited at the opening of the cervix (cervical AI).

In goat, commercially acceptable pregnancy rates (67.5%) have been reported in case of cervical AI with cooled diluted fresh semen [1,13,31,32], whereas with F-T semen these figures are much lower [33–35]. In Thailand, in small holdings, prenancy rates of 29% to 30% have been reported when goats were artifically inseminated using F-T semen [36]. However, the long distance between the semen production centers and the goat farms limits the use of cooled fresh semen [1]. In addition, continuous use of cooled fresh semen produced and used at the same farm enhances inbreeding in goat population [37]. Therefore, for genetic improvement of goats, use of AI with F-T semen seems to be essential [14,20]

The anatomy of cervix and the cervical penetrability of the crossbred meat goat have been reported previously [7]. During the follicular phase, length of cervix is 3.45±0.16 cm and 4.16±0.15 cm in nulli- and multi-parous goats, respectively, and parity had no effect on the number of cervical rings. Attempts have been made to relax the cervix by using exogenous hormones and administration of oxytocin [22,38], FSH, LH [39,40], PGE2 [23], PGE1 [39], and HA [26] also have been tried in sheep and/or goat with variable success rates. However, among these substances HA has been found to be the more promising one and topical application of low molecular weight HA, 48 hours after progesterone sponge removal has been shown to increase the penetrability of the ovine cervix beyond 3.0 cm [26], thus making it possible to deposit semen deeper into the cervix or even intra-uterine via the cervix. As a matter of fact, during peri-ovulatory period, HA accumulates in the interstices between collagen fibers leading to their loosening and dispersal [30] that relaxes the cervix and makes it possible to achieve a deeper penetration of the cervix.

In the present study, cervical insemination was performed in goats with F-T semen with or without prior topical application of the cervix with HA and pregnancy rates were compared between the two groups. The overall pregnancy rates were significantly higher with (63.3%) than without (36.0%) topical application of the cervix with HA. In goats, pregnancy rates of 29% to 30% have been reported after cervical insemination with F-T semen [36] which is in agreement with 36% obtained for the “without HA” group in our study.

In sheep, the depth of insemination influences the fertility, and lambing rates increase as the depth of insemination into the cervix increases [9,11,12,14]. The pregnancy rates were increased when F-T semen was deposited deeper into the cervical canal [41]. We believe that the significantly higher pregnancy rate obtained in the HA group was the result of a deeper deposition of semen into the cervix or even intrauterine in some animals.

Although depth of penetration has been related to the breed [42] and age of both sheep [9,41,42] and goats [14], the results of our study demonstrate it is also possible to increase the depth of penetration and inseminate comparatively deeper in goats with the prior topical application to the cervix with HA as has been reported previously in case of sheep [26].

The data in the present study suggest that the application of HA induced cervical relaxation in goats making it possible to deposit the semen deeper into the cervix and even into the uterus particularly in the multiparous goats. In the HA group, at first insemination 52.9% goats were inseminated intra-cervix and/or intra-uterine, and this proportion increased to 64.7% at the second insemination. Whereas, without HA, none of the goats were inseminated intra-cervix and/or intra-uterine at either the first or the second insemination. Therefore, the higher pregnancy rate observed in Group 1 could easily be attributed to the deeper deposition of semen achieved with HA application in this group.

The findings of the present study have shown that in both nulli- and multi-parous goats together 12/14 (85.7%) which were inseminated intra-cervix in the HA group became pregnant while 9/12 (75.0%) from the intra-uterine group became pregnant. The higher pregnancy rate achieved with intra-cervical (85.71%) compared to intra-uterine (75.0%) insemination could be simply a reflection of low numbers of animals in these groups. Nevertheless, higher pregnancy rates achieved in the “HA group” (66.3%) compared to the “without HA” group (36.0%) suggests that topical application of the cervix with HA increased the pregnancy rate by increasing the ease with which the cervix could be traversed in the goat resulting in a deeper deposition of semen.

The CAI is a comparatively simple insemination tech nique; it is easy to perform and the inseminating pipette and other equipment are available and affordable. Such attributes of CAI make it ideal for the wide-spread use of semen from genetically elite proven Billy goats. However, the pregnancy rates obtained after CAI with F-T semen are lower and not commercially acceptable. Results of the present study have shown that limitation of lower fertility with F-T semen and CAI can be overcome by topical application of the cervix with HA prior to insemination. We found that the pregnancy rate was increased from 36.0% (CAI) to 66.3% in case of prior application of HA. Although, higher fertility rate obtained in the HA goats can also be attributed to an increase in the percentage of animals that were inseminated intra-cervix or intra-uterus.

In conclusion, the results of the present study demonstrate that significantly higher pregnancy rates can be achieved in goats when inseminated with F-T semen 4 to 6 h after topical application of the cervix with HA. We believe that the commercially acceptable fertility thus achieved with F-T semen would be helpful to disseminate elite genes from the genetically superior Billy goats at a wider scale and therefore, be helpful to improve production of goat farms.

## Figures and Tables

**Table 1:** The pregnancy rate after cervical insemination using frozen-thawed semen with or without topical application of Hyaluronan (HA) in nulliparous and multiparous goats

Cervical insemination	Number (%) of goats pregnant		

	Nulliparous goats	Multiparous goats	Overall
With HA	17/26 (65.4%)^a^	21/34 (61.8%)^a^	38/60 (66.3%)^a^
Without HA	12/31 (38.7%)^b^	6/19 (31.6%)^b^	18/50 (36.0%)^b^
Total	29/57 (50.9%)	27/53 (50.9%)	56/110 (50.9%)
p-value	0.045	0.035	0.004

a,b The values with different superscripts within the columns or rows differ significantly.

**Table 2:** The number and percentage of nulli- and multi-parous goats according to the site of semen deposition within the reproductive tract at the first insemination (52 h after sponge removal)1)

Cervical insemination	The site of semen deposition at first insemination		

	Cervical os	Intra cervix	Intra uterus
Nulliparous goats			
With HA	18/26 (69.23%)^b^	8/26 (30.77%)^c^	0/26 (0.0%)^e^
Without HA	31/31 (100%)^a^	0/31 (0.0%)^e^	0/31 (0.0%)^e^
Multiparous goats			
With HA	16/34 (47%)^b^	6/34 (17.65%)^d^	12/34 (35.29%)^c^
Without HA	19/19 (100%)^a^	0/19 (0.0%)^e^	0/19 (0.0%)^e^
Total goats			
With HA	34/60 (56.67%)^b^	14/60 (23.33%)^d^	12/60 (20%)^c^
Without HA	50/50 (100%)^a^	0/50 (0.0%)^e^	0/50 (0.0%)^e^

1) Goats were inseminated with or without cervical topical application of hyaluronan (HA) at 48 h after the sponge removal.

a–e The values with different superscripts within the columns differ significantly (p≤0.05) within each category of goats (nulliparous, multiparous and total).

**Table 3:** The number and percentage of nulli- and multi-parous goats according to the site of semen deposition within the reproductive tract at the second insemination (56 h after sponge removal)

Cervical insemination	The site of semen deposition at second insemination		

	Cervical os	Intra cervix	Intra uterus
Nulliparous goats			
With HA	18/26 (69.23%)^b^	8/26 (30.77%)^c^	0^d^
Without HA	31/31 (100%)^a^	0^d^	0^d^
Multiparous goats			
With HA	12/34 (35.29%)^c^	10/34 (29.41%)^c^	12/34 (35.29%)^c^
Without HA	19/19 (100%)^a^	0^d^	0^d^
Total goats			
With HA	30/60 (50%)^b^	18/60 (30%)^c^	12/60 (20%)^d^
Without HA	50/50 (100%)^a^	0^e^	0^e^

HA, hyaluronan.

1) Goats were inseminated with or without cervical topical application of hyaluronan (HA) at 48 h after the sponge removal.

a–e The values with different superscripts within the columns differ significantly (p≤0.05) within each category (nulliparous, multiparous and total) of goats.
